# Acute Supplementation with Capsaicin Enhances Upper-Limb Performance in Male Jiu-Jitsu Athletes

**DOI:** 10.3390/sports10080120

**Published:** 2022-08-09

**Authors:** Bruno Victor Corrêa da Silva, Gustavo R. Mota, Moacir Marocolo, Jeffrey S. Martin, Luciano Sales Prado

**Affiliations:** 1 LAFISE–Exercise Physiology Laboratory, Federal University of Minas Gerais, Belo Horizonte 31310-250, Brazil; 2 Exercise Science, Health and Human Performance Research Group, Department of Sport Sciences, Institute of Health Sciences, Federal University of Triangulo Mineiro, Uberaba 38025-350, Brazil; 3 Physiology and Human Performance Research Group, Department of Physiology, Federal University of Juiz de Fora, Juiz de Fora 36036-900, Brazil; 4 Department of Basic Medical Sciences, DeBusk College of Osteopathic Medicine at Lincoln Memorial University—Knoxville, Knoxville, TN 37932, USA

**Keywords:** power output, ergogenic aids, sports nutrition, combat sports, capsaicin

## Abstract

The present study investigated whether acute capsaicin (CAP) supplementation improves mean power output (MPO) and peak velocity (PV) during the performance of the free bench press exercise (FBP). Twelve (*n* = 12) male Brazilian Jiu-Jitsu (BJJ) athletes (age: 24.3 ± 1.5 years, height: 1.74 ± 0.1 m, body mass: 75.7 ± 10.1 kg) participated in this randomized, placebo (PLA)-controlled, double-blind, crossover trial. For each condition, 45 min after CAP (12 mg purified) or PLA (12 mg of Celulomax E) consumption, the participants performed four sets of five repetitions of FBP at a load of 60% of body mass with five-min rest intervals. The MPO (t = 5.6, df = 11, *p* = 0.001, EF = 0.3, IC 95% = −0.55 to 1.05) and PV (t = 5.4, df = 11, *p* = 0.001, EF = 0.5, IC 95% = −0.32 to 1.30) were significantly higher with CAP supplementation versus PLA. Acute CAP supplementation appears to improve MPO and PV during FBP in male BJJ athletes.

## 1. Introduction

Capsaicin (CAP) (8-methyl-N-vanillyl-6-nonenamide) is the active component of chili peppers, belonging to the genus Capsicum [1]. Dietary CAP can elicit several metabolic effects, such as catecholamine secretion, glucose absorption, and thermogenesis [2]. Additionally, CAP has been shown to have anti-inflammatory, anti-cancer [3], anti-oxidant [4], analgesic [5], and anti-obesity properties [6].

Regarding exercise performance, acute CAP supplementation may have positive effects on endurance, resistance, and/or combined exercise [7]. A potential mechanism proposed that may explain its ergogenic effects on performance involves the modulation of the transient receptor potential vanilloid 1 (TRPV1) channel [8]. The activation of TRPV1 increases calcium release in the sarcoplasmic reticulum of muscle cells, increasing muscle force and fatty-acid oxidation and sparing glycogen, resulting in reduced muscular fatigue and, consequently, increased performance [9]. Furthermore, TRPV1 might have an analgesic effect that increases pain tolerance during exercise, which may attenuate declines in force production and/or increase volume in resistance training [1]. Lastly, TRPV1 could increase the release of acetylcholine, resulting in increased muscle power and increased endurance performance [7].

Power and velocity are mechanical parameters associated with performance in combat sports [10]. Specifically in Brazilian Jiu-Jitsu (BJJ), performance on power tests can explain the variations in the effectiveness of actions, as well as in chokes, joint locks, and takedowns [11]. In this sense, any muscle-power-output improvements from training and/or supplementation might contribute to improved performance in BJJ athletes [11].

To our knowledge, no study has yet investigated the acute effect of CAP supplementation on mean power output (MPO) and peak velocity (PV) during resistance-exercise training for the upper limbs. In the literature, the most common supplement used to induce acute ergogenic effects on power output and PV is caffeine [12]. However, caffeine can induce some adverse effects, such as diuresis, headache, dependence, mood shifts (e.g., irritability, anxiety, depression, etc.), and drowsiness [13]. Given that 12-millgram doses of CAP have been reported to be well tolerated in supplementation studies, with no reports of any “hot” sensations or gastrointestinal distress, CAP could be an interesting alterative to caffeine [14]. Given the importance of upper-limb performance in BJJ athletes, ergogenic aid from CAP supplementation may have practical and impactful applications for both training and competition. Therefore, we sought to determine whether acute CAP supplementation would improve MPO and/or PV during the FBP exercise in BJJ athletes. We hypothesized that acute supplementation of CAP would improve MPO and PV.

## 2. Materials and Methods

### 2.1. Experimental Design

**Figure 1:**
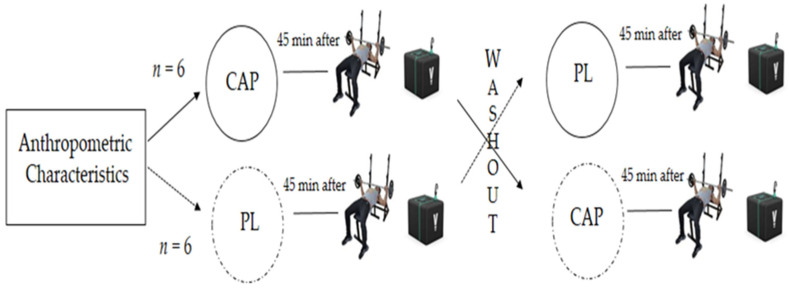
Experimental design of the study. After pilot, participants reported to the laboratory for anthropometric measurement and they were allocated one of two conditions, placebo (PL) or capsaicin (CAP). After one week of washout, the participants proceeded with the same assessments under the other condition.

A randomized, double-blind, placebo (PLA)-controlled, crossover experimental design was used in this study. Using a random number generator, each participant took part in two experimental trials at the same time of day and under laboratory-controlled conditions (21.4 ± 0.5 °C dry temperature; 30.5 ± 5.5% relative humidity). Experimental trials were separated by one week to allow complete recovery and CAP washout [15] (Figure 1). The subjects and investigators were blinded to the substances ingested and results of MPO and PV.

### 2.2. Participants

Twelve (*n* = 12) male Jiu-Jitsu competitors were recruited for this study. The anthropometric characteristics of athletes are presented in Table 1. The criteria used to classify the subjects as athletes were: a minimum of 3 years of BJJ training at a level ranging from purple to black belt; being a federated competitor; and having been a medalist (gold, silver or bronze) in national and/or international competitions of the BJJ Confederation (CBJJ) or International BJJ. Exclusion criteria included: (1) less than 2 years of resistance-training experience, (2) lack of familiarity with protocol of the study; (3) any medical contraindications that might interfere with the exercise protocol; (4) use of any ergogenic substance during the previous 3 months; (5) current smoker; and (6) injury that would limit performance during the study protocol.

The study was approved by the Ethics Research Group of the Federal University of Minas Gerais (protocol number: 24624919.3.0000.5149), the research was conducted according to the 2008 Revision of the Declaration of Helsinki, and all participants signed a consent form and were informed about the purpose of the study and the possible risks.

### 2.3. Experimental Protocol

At the first visit, prior to experimental trials, anthropometric characteristics were measured. Height was measured on a fixed stadiometer (Sanny, standard model, São Paulo, Brazil) and total body mass, muscle mass, fat mass, and body-fat percentage were measured using the Tanita-305 body-fat analyzer (Tanita Corp., Tokyo, Japan) following manufacturer’s guidelines.

Food questionnaires were distributed to all participants to record food and fluid intake for the 24 h preceding each trial. Participants were instructed to consume breakfast at home 2 h before each experimental trial, and to replicate the dietary intake during all the experimental trials. All food intakes were analyzed for total kilocalorie and macronutrient composition (Dietpro Version 5.8; Dietpro.com, Valencia, Spain) to ensure that dietary intake was similar between experimental trials. The software utilized the database of Brazilian food-composition tables to calculate dietary intake. The participants were instructed not to consume chili peppers or other spicy foods, as well as alcohol, and/or stimulant drinks, for 24 h prior to the assessment. During the study period, the participants were instructed not to use any other supplement or ergogenic substance, or to make changes to their regular diet. Mean caloric and macronutrient consumption in the 24 h prior to each condition are presented in Table 2.

For the experimental trials (first and second visits), each participant randomly consumed either the PLA (12 mg of Celulomax E) or 12 mg of purified CAP (Cosmed Manipulates^®^, Pedro Leopoldo, Brazil). This dosage was selected because other studies reported that 12 mg supplementation did not increase gastric motility [14,16]. Additionally, in our pilot study, we did not observe any cases of gastrointestinal discomfort following acute ingestion of a 12 mg CAP supplement. Identical, flavorless capsules were used for PLA and CAP and an independent assistant was assigned to deliver the supplements to every participant to ensure double-blinding. All capsules were consumed with water. PLA or CAP were taken in the laboratory and ingested 45 min prior to the experimental protocols [17]. This time period was used because the capsaicin peak in the blood occurs ~45 min after consumption, and complete plasma clearance occurs ~105 min after intake [17].

### 2.4. Protocol

Before the experimental trial, participants performed a brief warm-up of FBP with 2 sets of 5 repetitions with 40% of body mass (BM). Each set was separated by a 3 min rest period. After the warm-up, participants completed 4 sets of 5 repetitions, separated by 5 min of rest, of FBP at 60% of BM. The athletes were instructed to lower the bar (eccentric phase) in a controlled manner (2 s) until the barbell lightly touched their chest and then to move the load as quickly as possible (concentric phase) [18]. To ensure full range of motion, participants were instructed to hold the bar on their chest at the lowest point of the eccentric phase and to provide full extension during the concentric phase. A linear encoder measured the barbell’s movement distance (in centimeters) during each repetition. The MPO and intraclass correlation coefficient (ICC) were measured in the previous pilot study. The MPO in the FBP was determined through a second-order polynomial adjustment considering the mean relative loads used from 40% BM to 100% BM and the generated power output [19]. This alternative form of identifying optimal power load by BM has been recommended and used in the literature [20,21]. To measure mechanical parameters (i.e., MPO and PV), a linear position transducer (Vitruve^®^ Madrid, Spain) was attached to the barbell. This device was previously validated for MPO and PV measurements [22]. All athletes were familiarized with the testing procedures prior to testing due to their continuous assessments in their respective training routines.

### 2.5. Statistical Analyses

The data normality was verified using the Shapiro–Wilk test. Paired *t*-tests and corresponding effect sizes (ES) (Cohen’s d) were run to compare mean values of MPO and PV between conditions. Cohen’s d values were defined as: <0.2 -trivial; >0.2 small; >0.5 moderate; and >0.80, large [23]. A 2-way repeated-measures analysis of variance (2 (condition) × 4 (sets)) with Bonferroni adjustment for multiple comparisons was used to compare performance across the sets. When a significant interaction was observed, a Bonferroni post hoc test was applied. The Standard Error of Measurement (SEM) was calculated with the following formula: SEM = SD√ (1 − ICC). The smallest worthwhile change (SWC) was calculated as SWC = SEM × 1.96 × √2 [24]. All data are presented as mean ± SD or overall mean (bars) and individual performance (lines). Significance was set at *p* ≤ 0.05 a priori.

## 3. Results

**Figure 2:**
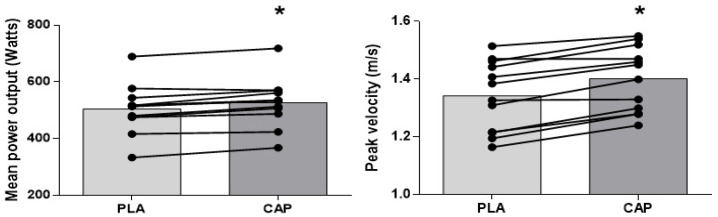
Comparison between effect of acute placebo (PLA) and capsaicin (CAP) ingestion on mean power output (**left**) and peak velocity (**right**) across 4 sets of 5 repetitions of free bench press at 60% of body mass. Data are presented as overall mean (bars) and horizontal lines represent individual data. * = statistically significant difference between CAP and PLA conditions.

Figure 2 presents mean MPO and PV measurements across all sets. The MPO was significantly higher (t = 5.6, df = 11, *p* = 0.001, EF = 0.3, IC 95% = −0.55 to 1.05) in the CAP (527 ± 85 W) compared with the PLA condition (504 ± 86 W). CAP supplementation resulted in increases in MPO above the SWC (12 W) of the PLA in 83% of individuals. The mean of the PV of all sets was significantly higher (t = 5.4, df = 11, *p* < 0.001, EF = 0.5, IC 95% = −0.32 to 1.30) in the CAP (1.40 ± 0.11 m/s) compared with the PLA condition (1.34 ± 0.12 m/s). CAP supplementation resulted in increases in peak velocity above the SWC (0.07 m/s) of the PLA in 83% of individuals. In comparison to PLA, the pre-exercise ingestion of CAP enhanced MPO and PV during the FBP 4.4% and 4.3%, respectively.

**Figure 3:**
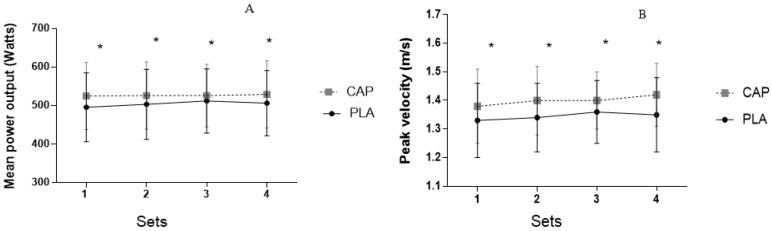
Comparison between effect of acute placebo (PLA) and capsaicin (CAP) ingestion on mean power output (**A**) and peak velocity (**B**) during 4 sets of 5 repetitions of free bench press. * = statistically significant difference between PLA and CAP conditions.

Figure 3 illustrates the comparison between CAP and PLA conditions on MPO and PV for each set. CAP condition was significantly higher compared to each respective set for the PLA condition. There was a significant main effect of condition for MPO (F = 30.886, *p* = 0.001) and PV (F = 43.238, *p* = 0.001), but not an interaction for either MPO (F = 0.848, *p* = 0.49) or PV (F = 1.142, *p* = 0.34).

## 4. Discussion

The purpose of this investigation was to evaluate the ergogenic effects of an acute dose of CAP supplementation in MPO and PV on upper-body resistance exercise in BJJ athletes. Our main finding was that a 12 mg dose of CAP acutely increased MPO and PV compared with PLA during FBP exercise.

To the best of our knowledge, this is the first study to investigate the acute effects of CAP supplementation on MPO and PV during upper-body resistance exercise with a protocol specific for muscle power. There is a body of evidence in the literature reporting the acute ergogenic effects of caffeine [12], citrulline [25], and beetroot juice [26] on peak power or peak velocity during upper-body resistance exercise. However, the majority of these studies did not use conventional power training. In the studies on supplementation with caffeine and citrulline, incremental power-load tests were employed, and the exercises were performed to failure with a high number of repetitions (~15 on average). Traditionally, training for power is performed with a lower number of repetitions to minimize the decrease in mechanical parameters during sets [27]. When sets are performed with high levels of fatigue, neuromuscular adaptations are compromised [28]. In the study with beetroot-juice supplementation, the protocol was more characteristic of power training [26], but the numbers of sets and repetitions were low (2 × 2 repetitions) and the intensity (70% 1RM) was above the recommendations for power training for FBP in BJJ athletes [19]. Additionally, beetroot juice is not classified as a stimulant supplement, whereas caffeine and CAP are.

In the present study, the acute ingestion of CAP increased the MPO and PV by 4.4% and 4.3%, respectively, in comparison with PLA. The acute effect of caffeine on the mechanical variables (power output and velocity) has a reported range of increase from 5 to 15% [29]. With regards to effect size, we also observed a small effect (d = 0.3 to 0.5), which was similar to the values reported with other supplements used to increase acute performance [25]. For example, caffeine exerts effects of similar magnitude (d = 0.21) on power performance. Despite their relatively modest effect size, these supplements may offer meaningful benefits to athletes during training.

The potential mechanisms behind the improvements in MPO and PV by CAP may be related to TRPV1 activation, which increases the availability of free calcium, leading to greater interaction between actin and myosin filaments and potentially enhancing force production [7]. Previous evidence using both human and animal models provided a rationale for this premise. In mice, CAP enhanced calcium release from the sarcoplasmic reticulum, increasing the force output of fast-twitch fibers [30]. Additionally, it has been reported that calcium release by the sarcoplasmic reticulum increases force production through post-activation potentiation (PAP) [31]. Other possible mechanisms could include the action of CAP in the TRPV1 present at the neuromuscular junction, where CAP can induce presynaptic modulation, leading to an increase in the evoked acetylcholine release [7]. Notably, the mechanisms of action were not determined herein and require further investigation.

Our results are in accordance with previous investigations relating ergogenic effects to acute CAP supplementation [7]. In rodents, acute CAP supplementation increased swimming time to exhaustion [32] and grip strength [30]. In humans, CAP ingestion acutely improved volume-load in resistance exercise [14], decreased 1500 m running time-trial times [16], increased the time to exhaustion during a high-intensity intermittent (HIIT) protocol [33] and improved subsequent resistance-exercise performance in resistance-trained men after HIIT [17]. The results of these studies together suggest that acute CAP supplementation may attenuate some of the fatigue interference in endurance and resistance exercise, and increase MPO and PV during upper-limb exercise.

This study has some limitations. We used a single dose that was not relative to body mass. However, the ideal dose of CAP to induce ergogenic effects is not yet clear and we used the same dosage as previous studies [14,17]. The study was also limited by its small sample of male BJJ athletes; hence, our results cannot be extrapolated to more specialized populations (e.g., master BJJ athletes). Additionally, the ergogenic effects found here were specific to FBP only, and did not directly measure BJJ performance; therefore, their extrapolation to performance may be speculative.

The results of our study could help nutritionists, coaches, and athletes with nutritional strategies, as well as with finding a supplement that could improve power and velocity in BJJ athletes. Power and velocity are crucial mechanical parameters associated with better performance in combat sports. Muscle power is associated with faster movements, which allow grapplers to attack and defend more quickly against resisting opponents during BJJ matches. Thus, beyond regular training and recovery, the results of this study suggest that acute CAP supplementation can be used as a nutritional strategy to acutely increase mechanical parameters (i.e., PP and PV) in upper-limb exercise in BJJ athletes. Future studies are necessary to investigate the acute effects of CAP on other variables of resistance training, such as volume, as well as the chronic effects of CAP on mechanical parameters and their relationships with power training.

## 5. Conclusions

Acute CAP supplementation 45 min prior to training improves the MPO and PV of consecutive bouts of upper-limb, FBP resistance exercise performed in accordance with power development in male BJJ athletes. These findings suggest that 12 mg of CAP ingested prior to exercise is well tolerable [14,16], and may be of benefit for BJJ athletes seeking to enhance upper-extremity muscle power.

## Figures and Tables

**Table 1:** Anthropometric characteristics of athletes (*n* = 12).

Variables	Mean ± SD
Age (years)	24.3 ± 1.5
Height (m)	1.74 ± 0.1
Total body mass (kg)	75.7 ± 10.3
Muscle mass (kg)	64.5 ± 6.5
Fat mass (kg)	8.2 ± 3.9
Body fat (%)	10.2 ± 3.6

**Table 2:** Dietary intake and macronutrient distribution 24 h before each trial.

Macronutrient	Placebo (*n* = 12)	Capsaicin (*n* = 12)	*p*-Value
Carbohydrate (g)	240 ± 99	242 ± 98	0.96
Protein (g)	204 ± 74	200 ± 84	0.91
Lipid (g)	81 ± 38	83 ± 39	0.90
Total intake (kcal)	2469 ± 883	2518 ± 876	0.89
Carbohydrate (g kg^−1^)	3.2 ± 1.1	3.2 ± 1.2	0.11
Protein (g kg^−1^)	2.6 ± 0.6	2.6 ± 0.8	0.60
Lipid (g kg^−1^)	1.1 ± 0.4	1.1 ± 0.5	0.10
Total intake (kcal kg^−1^)	32.2 ± 9.1	32.9 ± 9.0	0.60

Data are mean ± SD.

## Data Availability

The data presented in this study are available on request from the corresponding author. The data are not publicly available due to privacy.
